# Structural Pattern Differences in Unbranched Rod-Like RNA of Hepatitis Delta Virus Affect RNA Editing

**DOI:** 10.3390/v11100934

**Published:** 2019-10-11

**Authors:** Chao-Wei Hsu, Horng-Heng Juang, Chien-Yi Kuo, Hsin-Pai Li, Shan-Bei Iang, Siao-Han Lin, Chau-Ting Yeh, Mei Chao

**Affiliations:** 1 Liver Research Center, Department of Hepato-Gastroenterology, Chang Gung Memorial Hospital, Guishan, Taoyang 33302, Taiwan; hsu2406@cgmh.org.tw (C.-W.H.); chauting@cgmh.org.tw (C.-T.Y.); 2 Department of Anatomy, Chang Gung University, Guishan, Taoyang 33302, Taiwan; hhj143@mail.cgu.edu.tw; 3 Department of Microbiology and Immunology, Chang Gung University, Guishan, Taoyang 33302, Taiwan; strangertue@mail.cgu.edu.tw (C.-Y.K.); paili@mail.cgu.edu.tw (H.-P.L.); isbisb@mail.cgu.edu.tw (S.-B.I.); ms00234@hotmail.com (S.-H.L.); 4 Division of Microbiology, Graduate Institute of Biomedical Sciences, Chang Gung University, Guishan, Taoyang 33302, Taiwan

**Keywords:** hepatitis delta virus, RNA editing, delta antigen, ADAR1

## Abstract

Hepatitis delta virus (HDV) RNA forms an unbranched rod-like structure and complexes with the delta antigen (HDAg). Host ADAR1-catalyzed RNA editing at the amber/W site of the small HDAg leads to the production of the large HDAg, which inhibits replication and is required for virion assembly. For HDV genotype 1, amber/W editing is controlled by HDAg and the RNA structure immediate vicinity and downstream of the editing site. Here, the effects of 20 mutants carrying an increased length of consecutive base-pairing at various sites in HDV RNA on amber/W site editing were examined. All nine mutants carrying genomic regions that formed up to 15 consecutive base pairs, which is also the maximum length observed in 41 naturally occurring HDV genomes, showed normal editing rate. However, mutants carrying a 16 or 17 consecutive base-paired antigenomic segment located as far as 114 nt upstream could increase editing efficiency, possibly by interfering with HDAg binding. These data show for the first time that extended base-pairing upstream of the amber/W site could increase HDV RNA editing efficiency. Furthermore, it appears that the naturally occurring HDV RNA structures have been selected for suboptimal amber/W RNA editing, which favors the HDV replication cycle.

## 1. Introduction

Hepatitis delta virus (HDV) virion is composed of hepatitis B virus surface antigens (HBsAgs) surrounding a ribonucleoprotein (RNP) complex that comprises the circular RNA genome and multiple copies of the delta antigen (HDAg) [1,2,3]. The latter is the sole HDV-encoded protein and occurs as small and large forms. The circular RNA genome of HDV comprises approximately 1700 nucleotides (nt), making it the smallest genome known to infect humans [3,4]. The HDV genome and its replication intermediate, the antigenome, both form a characteristic unbranched rod-like structure, in which ~70% of the nt form base pairs (bp). Numerous HDV isolates from around the world have been sequenced and at least eight genotypes (clades), HDV-1~8, have been defined [5,6]. Although the isolates can differ in sequence by as much as 40%, the unbranched rod-like structure of the HDV RNA is largely maintained [6]. During replication, ~0.8-kb antigenomic-sense mRNA encoding small HDAg is produced, which is required for HDV RNA synthesis. The host ADAR1 edits the full-length antigenome RNA by deaminating the adenosine within the amber stop codon (UAG) to inosine and thereby altering the codon to UGG, which encodes tryptophan (W). This editing site is thereby called the amber/W site [7,8,9], and the above-described editing extends translation by an additional 19 or 20 amino acids to yield large HDAg, which inhibits genome replication and is required for packaging of the virus [10,11]. The regulatory mechanisms of editing at the amber/W site have been experimentally evaluated for in HDV-1 and HDV-3. Editing of HDV-1 involves the unbranched rod structure and is inhibited by HDAg [9,12]. HDAg specifically binds the HDV RNA unbranched rod structure [13], which might prevent access to ADAR1 [14,15]. The amber/W site of HDV-1 occurs as an A-C mismatch in the midst of eight canonical Watson–Crick bp [16,17]. Improved base-pairing, particularly in the region 15–25 nt downstream of the editing site, has been shown to significantly increase HDV-1 editing [14,18]. The role of sequences upstream of the amber/W site of HDV-1 has not been examined in detail. Base-paired editing of HDV-3 amber/W site is not inhibited by HDAg and involves a rearranged double-hairpin structure (nearly 80 bp) flanking a central base-paired region that includes the amber/W site, which forms an A-U pair [19].

As the HDV replication cycle depends heavily on structural features of its RNA, the present study was undertaken to explore how differences in the patterns of the predicted HDV-1 unbranched rod-like structure could affect HDV-1 RNA editing. We created small (1–5 nt) deletions, insertions and/or base substitutions at various sites located opposite the HDAg coding region, which serve primarily to form the unbranched rod-like structure, with the goal of removing predicted unpaired bases, and consequently, increasing the length of consecutive base-pairing in the HDV RNA. Our findings indicate that extended base-pairing upstream of the amber/W site could increase editing efficiency, possibly by interrupting the interactions between the HDV RNA and HDAg. Based on these results, we propose a model explaining how the regulation of amber/W editing of HDV-1 is mediated.

## 2. Materials and Methods 

### 2.1. Plasmids

**Figure 1:**
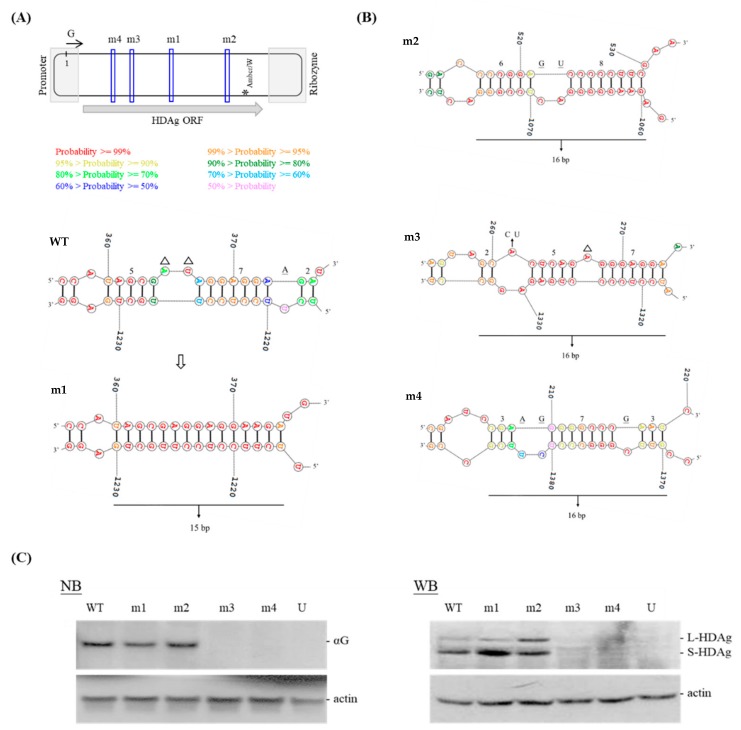
Hepatitis delta virus (HDV) m1~m4 mutants. (**A**,**B**) Schematic representation of HDV RNA and the design of the HDV mutants. The oval indicates the HDV genome sequence, and the arrow represents the genomic (G) RNA orientation. The upstream portion of the open reading frame (ORF) encoding HDAg is located within one end of this structure. The sequence is highly heterogeneous among the HDV genotypes [5,6] and is likely to act as a promoter for the initiation of RNA-directed RNA synthesis [30]. During rolling-circle replication, the genome and antigenome are both autocatalytically cleaved by self-encoded ribozymes. For this, they fold into alternative pseudo-knotted structures located at one end of the HDV RNA, in a region that represents the most highly conserved region of the HDV RNA [31,32]. These ORF-opposite sequences serve primarily to form the unbranched rod-like structure and thus provide an excellent target for introducing mutations into the HDV genome. The blue boxes on the HDV genome denote the regions at which the m1~m4 mutations were introduced. Star indicates the amber/W editing site. Secondary structures for HDV WT and the m1 mutant were predicted using RNA structure (version 6.0.1) [23,24] and partial unbranched rod-like RNA structures of the genomic HDV RNAs containing the mutation sites are shown. Color annotation was performed according to base-pairing probability; more-probable pairs are more likely to be correctly predicted than less-probable pairs [33]. For example, our base-pairing probability data indicate that we had correctly predicted the secondary structure of the m1 mutant. Base insertions and deletions are underlined and indicated with triangles, respectively. Base substitutions were shown above the arrow and vertical lines indicate the formation of base-pairing in the HDV circular genome. (**C**) Accumulation of HDV RNA and HDAg in Huh-7 cells transfected with mutants m1~m4. αG represents antigenomic RNA. Actin protein and mRNA levels were determined to compare the total protein and RNA levels in each lane, respectively.

The pGEM-4Z-D1I contained an XbaI-XbaI (nt 781) monomer cDNA insert representing an HDV-1 sequence of Italian origin (clone I; GenBank accession number M21012) [20,21]. pGEM-4Z-D1I and its related constructs were used as the templates for single-step, single-primer PCR-based mutagenesis [22]. We designed 20 mutants carrying different patterns of unbranched rod-like RNA structure (Figure 1A,B and Figures S1 and S2). The HDV unbranched rod-like RNA structures of WT and the mutants were predicted using RNA structure (version 6.0.1) [23,24]. The mutations were introduced into a region covering nt ~100–580. m2~m7 contained a segment of 16 consecutive bp at various regions on the HDV genome. The m1 and m8~m14 mutants had elongated regions of up to 15 bp. m2-15, m2-16, and m2-17 were designed from m2. The other three mutants, m-17a, m-17b, and m-17c, carried 17 consecutive bp located 11 nt, 76 nt, and 114 nt 5′ of the amber/W site, respectively. The primer sequences and templates used for single-step, single-primer PCR-based mutagenesis [22] are summarized in Table S1. The primer names are consistent with those of the resulting plasmids carrying the mutated sequences. These PCR-generated mutants were confirmed by sequencing. To construct HDV genome-expressing plasmids, we used a standard molecular cloning protocol involving restriction enzyme digestion and ligation reactions [25]. Briefly, the XbaI-digested HDV cDNA monomers of pGEM-4Z-D1I and its derivative mutants were subcloned into the pCR3.1 T-vector (Promega, Madison, WI, USA), which contains a human cytomegalovirus immediate-early promoter. Clones carrying the HDV head-to-tail cDNA dimer in a proper orientation were collected. All of the generated plasmids expressed genomic HDV RNA for the subsequent RNA-directed RNA synthesis of HDV.

The inserts of the nonreplicating HDV amber/W editing reporter constructs, pCR3.1-WTNR and pCR3.1-m-17cNR, were obtained by PCR amplification of a region covering nt 216–1381 using HDV cDNA dimer constructs expressing WT and m-17c mutant sequences, respectively, as the template [15]. Total RNA samples were extracted three days post-transfection in cells transfected with amber/W editing reporter plasmids pCR3.1-WTNR and pCR3.1-m-17cNR.

### 2.2. DNA Transfection and Posttransfection Analyses of HDV RNA and HDAg

Huh-7 cells were cultured in 35-mm dishes and transfected with 4 μg of DNA per dish using Lipofectamine (Invitrogen, Carlsbad, CA, USA), as described elsewhere [21,26]. Six days after cells were transfected with m1~m14, intracellular HDV, antigenomic RNA and the two forms of HDAg (as markers for HDV replication) were analyzed by Northern blotting (NB) and Western blotting (WB) analyses, respectively. These procedures were performed as previously described [21,26], except that DIG Easy Hyb (Roche, Basel, Switzerland) was used to visualize the results of the NB analyses. All transfection experiments were repeated between two and four times, and representative results are shown. 

To analyze the amber/W editing, total RNA samples were extracted from the transfected cells at 12 days post-transfection. Residual plasmid DNA was removed by digestion with RQ DNase I (Promega), the RNA was reverse transcribed using oligo(dT) [27], and the obtained cDNA was subjected to PCR using previously published primer pair 18/55′ [26]. The obtained PCR products were sequenced using primer 18, and the un-edited versus edited HDV RNA ratio was estimated.

Huh-7 cells were also co-transfected with pS1X, which encodes HBsAg [10], and the HDV genome expression plasmids, WT and m2 mutants, to produce HDV virions. Pelleting and analyses of HDV virions were as mentioned previously [28].

## 3. Results

### 3.1. Effect of Structural Differences in the HDV Replication

The unbranched rod-like RNA structure, comprising short base-paired segments interspersed with small bulges, plays important roles in the HDV replication cycle [4,20]. However, the longest double-stranded segment in the computer-predicted RNA structure of the HDV genome, which is the RNA species that enters a cell to initiate RNA replication, had not been systemically examined. We downloaded 41 HDV-1 isolates from GenBank and used RNA structure [29] to predict their secondary structures. As summarized in Table 1, the longest consecutive base-paired regions ranged from nine to 15 bp in length. Most of the analyzed sequences (78%) had a genomic segment containing 10–12 consecutive bp. Three had a genomic segment carrying the maximum observed base-pairing of 15 consecutive bp. These observations prompted us to design and analyze a series of mutants with different base-pairing patterns in the predicted rod-like genome of HDV-1 clone I sequence [20], which includes numerous short (2 to 11 nt) base-paired segments interspersed with small bulges. We designed HDV mutants by introducing small insertions, deletions, and/or point mutations to remove the bulges and increase the base-pairing at different domains on HDV genome, which created structures with higher stability than that of the wild-type (WT). For example, nt 360–375/1217–1231 were predicted to form three short base-paired regions (5, 7, and 2 bp) interrupted by two small bulges (Figure 1A, bottom). m1 was designed to increase the consecutive bp to 15 by destroying these two bulges. The free energies of the genomic RNA of the WT HDV and m1 mutant were −880.7 and −890.1 kcal/mol, respectively. Furthermore, the base-paired patterns upstream and downstream of the improved base-paired segment remained unchanged. Similar prediction results were obtained for all of the mutants designed in this study. Since 15 bp was the maximum length seen in the predicted structures of naturally occurring HDV RNA (Table 1), we constructed HDV mutants m1~m4 to form segments with 15 (m1) or 16 (m2~4) consecutive bp (Figure 1B) on the HDV genome. We transfected Huh-7 cells with HDV genome-expressing plasmids, harvested RNA and proteins six days post-transfection, and analyzed the samples using NB and WB analyses, respectively. As shown in Figure 1C, m1 and m2 were replication-competent, whereas m3 and m4 failed to replicate. Surprisingly, m2, whose mutated domain (nt 516–529/1060–1075) mapped to ~50 nt upstream of the antigenomic amber/W editing site, exhibited an increase in the ratio of large HDAg to total HDAg (7% for WT versus 32% for m2) (Figure 1C).

We examined sequence reversion and/or the accumulation of more mutations using RNA isolated from cells six days post-transfection. Sanger sequencing of the RT-PCR product of a region covering 389–1310 nt indicated that no additional sequence change, other than at the amber/W site, compared to the original mutated sequence of m1 and m2. Sequencing of 40 cloned PCR products of m2 revealed no reversion and several base changes, but they were randomly distributed, and most were detected in only one clone.

**Figure 2:**
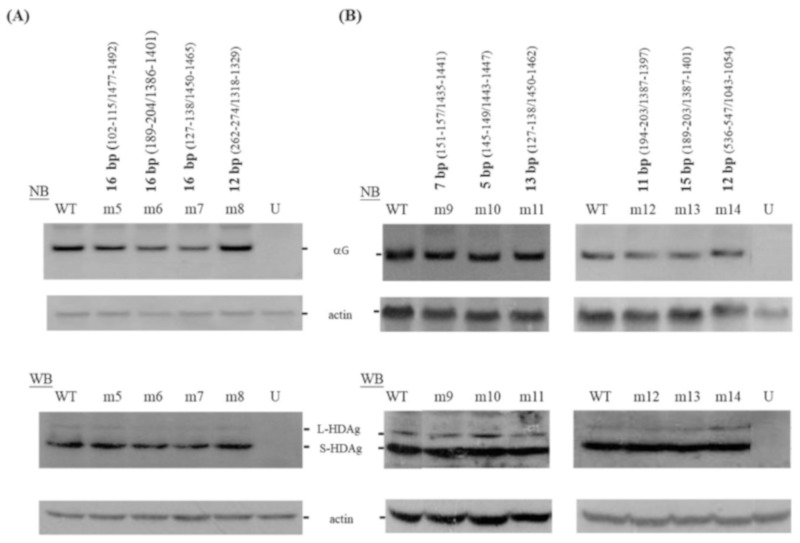
HDV mutants m5~14. The locations and length of the base-paired regions are summarized in Figure S1 and above the figures. Analyses of HDV RNA and HDAg in transfected Huh-7 cells are as described for Figure 1. The ratio of large HDAg to total HDAg for WT and mutants was consistently less than 8%. Lane U, untransfected control. The data for m9~11 were obtained from the same gel, while those for m12~14 were from a different gel.

We then designed three more mutants, m5~7, which each carried 16 consecutive bp on their genomes (Figure S1). We also designed mutant m8, in which the 16 consecutive bp of replication-incompetent m3 were reduced to 12 consecutive bp (Figure S1). As shown in Figure 2A, our post-transfection analyses indicated that the HDV RNA accumulation of m8 was similar to that of WT HDV, whereas this accumulation was only ~55–71% of the WT levels for m5~7. Thus, all of the six mutants constructed carrying 16 consecutive bp on the HDV genome behaved differently from WT. Two were replication-incompetent (m3 and m4), three had reduced replication abilities (m5~7), and one expressed more large HDAg (m2) compared to WT day 6 post-transfection.

We also selected regions in which base-paired segments were interrupted by asymmetric single-nucleotide bulges or symmetric bulges containing one unpaired nt on each side of the rod-like HDV RNA. Single-base substitutions, deletions, or insertions were used to remove each bulge and allow extended base-pairing. The sites at which m9~14 were mutated are presented in Figure S1. As shown in Figure 2B, all of the mutants were replication-competent and showed HDV RNA and HDAg levels similar to those of WT HDV. These data suggest that HDV can tolerate one or two experimentally designed nt changes that improve the base-pairing of regions in the unbranched rod-like genome to as many as 15 consecutive bp.

### 3.2. Kinetics of HDV Mutant m2 

**Figure 3:**
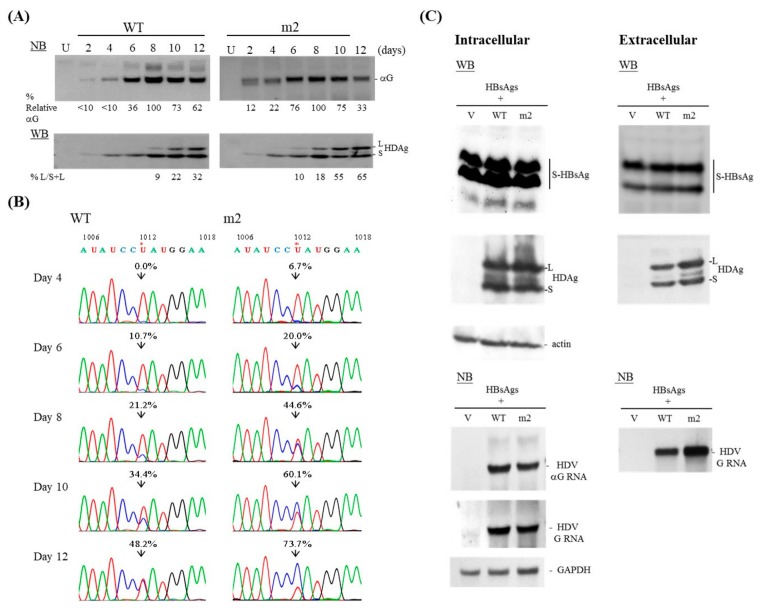
RNA replication and RNA editing of WT and m2 mutant HDV. (**A**) Kinetics of HDV RNA synthesis and HDAg expression in Huh-7 cells transfected with expression plasmids encoding the WT or m2 HDV genomes. RNA and proteins were harvested at the indicated times post-transfection and analyzed by NB and WB, respectively. (**B**) Validation of HDV RNA editing. RNA samples collected at various time points were subjected to RT-PCR followed by direct sequencing of a region containing the amber/W editing site. Shown is a sequencing chromatogram spanning 1006–1018 nt. Arrowheads indicate the position of the amber/W editing site. The percentage of “C” residue in the genomic RNA arising from editing of the antigenome was calculated and is presented above the arrowhead on each sequencing chromatogram. (**C**) Virion assembly of m2 mutant HDV. Intracellular RNA and proteins were harvested at 12 days post-transfection and analyzed by NB and WB, respectively. RNA loading was monitored by a GAPDH mRNA probe. HBsAg-packaged particles were concentrated from media collected on days 6–12 post-transfection. Lane V represents an experiment in which cells were co-transfected with vector plus the HBsAg expression plasmid. S-HBsAg, small form of HBsAg.

The study shown in Figure 1C is the first to suggest that the extension of base-pairing upstream of the editing site also affects HDV amber/W editing. We then performed a time-course analysis for WT and m2 HDV, every two days from day 2 to day 12 post-transfection. As presented in Figure 3A, NB analysis revealed that both WT and m2 accumulated HDV RNA, and that this reached a peak on day 8 and then declined. Our WB data indicate that small HDAg was barely detectable at day 2 and reached a peak on day 8 for both WT and m2. Thereafter, the level of small HDAg remained constant for WT but declined for m2. Large HDAg was detectable on day 6 and day 4 for WT and m2, respectively, and increased steadily thereafter in both cases until the last day of sample collection. About 32% and 65% of the total HDAg proteins were present as the large form in Huh-7 cells transfected with WT and m2, respectively, on day 12, as determined by WB. These WB data were consistent with our NB data showing that the HDV RNA level had dropped more significantly for m2 than for WT HDV at 12 days post-transfection, possibly due to the decreased level of small HDAg and the inhibitory effect of accumulated large HDAg in cells expressing the m2 mutant. The presence of RNA editing was further validated using RT-PCR amplification and Sanger sequencing (Figure 3B). Consistent with the data obtained from our WB experiments (Figure 3A), the sequencing results indicate that m2 underwent editing more efficiently than WT at all analyzed time points. For example, 48.2% and 73.7% of the RNA was found in the edited form in cells transfected with WT and m2 HDV, respectively, on day 12. The observed editing of WT HDV was dependent not only on ADAR editing, but also on the replication and accumulation of the altered antigenomic RNAs. However, the levels of large HDAg and the edited genome of m2 kept increasing even when the levels of small HDAg and HDV RNA replication declined significantly at 12 days post-transfection. Therefore, the structural features of m2 appear to play an important role in the increased editing rate. 

### 3.3. Packaging Ability of the m2 Mutant

As large HDAg is known to be required for HDV virion assembly [10], we next tested whether the increased RNA editing and subsequent upregulation of large HDAg associated with m2 yielded improvement in the packaging efficiency. Toward this end, we co-expressed HDV genomes and HBsAg expression constructs in Huh-7 cells. To examine the intracellular status, we assessed HDV RNA replication and viral protein expression in cell lysates on day 12 post-transfection. To examine extracellular components, we collected media from day 6 to day 12 post-transfection, concentrated the virions by centrifugation through a sucrose cushion, and dissolved the pelleted particles. As shown in Figure 3C, m2 released more HDV virions into the medium than WT HDV, as judged by the amounts of HDAg and HDV genomic RNA in the pelleted particles. The estimated difference between the genomic RNA of m2 mutant and WT HDV was 1.8-fold.

### 3.4. The Length, But Not the Position, of the Consecutive bp on the HDV Antigenome Affects RNA Editing 

**Figure 4:**
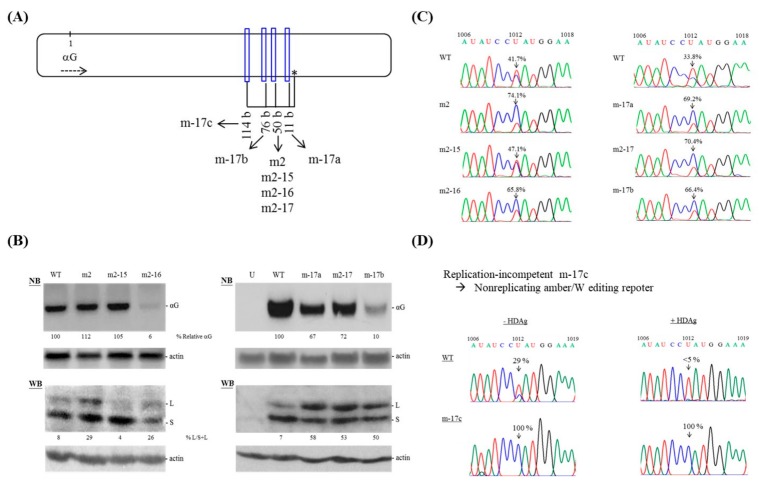
HDV mutants forming 15~17 consecutive bp upstream of the amber/W site. (**A**) Schematic representation of the locations of the HDV mutants on the HDV antigenome. Symbols and description are as described for Figure 1A, except that the HDV antigenome sequence was shown here. (**B**) Effect of mutations on HDV RNA accumulation and HDAg expression. Analyses of HDV RNA and HDAg are as described for Figure 1C. (**C**) Validation of HDV RNA editing is as described for Figure 3B. (**D**) Effect of the m-17c mutation on HDV RNA editing. HeLa cells were transfected with nonreplicating amber/W editing reporter constructs in the absence (- HDAg) or presence (+ HDAg) of a small HDAg-expressing plasmid. RNA samples were collected three days post-transfection. Shown is a sequencing chromatogram spanning 1006–1019 nt.

Among the replication-competent mutants containing a region of 16 consecutive bp on the genomic RNA, only m2 exhibited significantly increased amber/W site editing. Closer examination of the base-paired patterns of the mutants revealed that G-U bp were present in the elongated consecutive base-paired genomic regions of m4, but not in that of m2 (Figure 1B). A G-U pair in the genomic RNA would cause a C-A mismatched pair in the antigenomic RNA. Meanwhile, m2 carried a segment containing 17 constitutive bp on its antigenome due to the formation of an additional G-U bp between nt 515 and nt 1076 (Figure S2). To gain a better understanding of how the length of the consecutive bp could affect amber/W site editing, we designed mutants m2-15 and m2-16 (Figure 4A and Figure S2), which created a 15-bp and 16-bp region on the HDV antigenome, respectively. As shown in Figure 4B, m2 and m2-15 showed replication levels similar to that of WT, while m2-16 replicated inefficiently at day 6 post-transfection. WB data indicate that m2, but not m2-15, had an increased level of large HDAg expression relative to that of the WT HDV clone. Consistent with the dramatic inhibition of RNA synthesis seen for m2-16, this mutant was associated with downregulation of the expression of small HDAg. Surprisingly, m2-16 expressed a substantial level of large HDAg (Figure 4B). Data obtained using RT-PCR and subsequent direct sequencing further indicate that m2 and m2-16 exhibited increased editing efficiency compared to WT, whereas m2-15 did not (Figure 4C). Taken together, our results show that RNA editing is promoted by the presence of a segment that forms 16 or 17, but not 15, consecutive bp located 50-nt 5′ to the amber/W site.

To evaluate how the position of 17 consecutive bp upstream of the amber/W site affected RNA editing, we designed mutants with 17 consecutive bp located 11 nt (mutant m-17a), 50 nt (m2-17) and 76 nt (m-17b) 5′ of the amber/W site (Figure 4A and Figure S2). Each carried a region of 17 consecutive bp on both the genome and antigenome. As shown in Figure 4B, all three mutants were replication-competent, though to a lesser degree than the WT. Interestingly, WB analysis revealed that more than 50% of the HDAgs produced by the three mutants were large HDAg. Data obtained from direct sequencing of the PCR products further confirmed that all three mutants with 17 consecutive bp located at various regions upstream of the amber/W site exhibited increased editing efficiency compared to WT (Figure 4C). Similar to the data obtained from mutant m2, no reversion or additional sequence change other than at the amber/W site were observed in a region covering 389–1310 nt. Taken together, these data indicate that improved base-pairing in the region upstream of the amber/W editing site increased RNA editing.

### 3.5. HDAg Fails to Regulate Amber/W Editing of HDV Mutants Carrying Elongated Base-Pairing

An additional mutant, m-17c, carrying a region of 17 consecutive bp located further upstream at 114 bp upstream of the amber/W site (Figure 4A and Figure S2) was replication-incompetent (data not shown). We hypothesized that amber/W editing of this mutant is too efficient to produce enough small HDAg to support HDV RNA replication. To test this hypothesis, the previously established non-replicating RNA editing reporter system was employed here [15]. We introduced a HDV subgenome covering 216–1381 nt of the WT and m-17c mutant sequences into the expression construct, pCR3.1, which upon transfection of cultured cells produces a HDV antigenomic RNA that does not replicate or produce HDAg, but does serve as a substrate for editing [15]. Total RNAs were harvested three days post-transfection and editing at the amber/W site was quantified by direct sequencing of RT-PCR amplification products. Editing at the amber/W site was 29% for the WT sequence but reached 100% for the m-17c mutant sequence (Figure 4D, left). Given that the numerous bulges in the unbranched rod-like structure are essential for HDAg binding and such binding plays a crucial role in the suppression of RNA editing efficiency [18,34], we hypothesized that the elongated base-paired region in the m-17c mutated genome disfavors HDAg binding and thus promotes amber/W RNA editing. To test this, we co-transfected cells with small HDAg and WT or m-17c non-replicating constructs and examined amber/W editing. Indeed, the amber/W RNA editing of the WT sequence, but not the m-17c sequence, was inhibited in the presence of small HDAg (Figure 4D, right). Overall, our results support the notion that the naturally occurring HDV RNA is sub-optimal for amber/W editing and that HDAg is unable to control amber/W RNA editing on a mutated HDV antigenome containing an elongated double-stranded region, possibly because the mutation interferes with the binding of HDAg to the HDV RNA.

## 4. Discussion

The ADAR1-mediated amber/W editing regulates HDV RNA synthesis and virion assembly. For HDV-1, this amber/W site occurs as an A-C mismatch pair in the midst of 8 bp, and that increased base-pairing in the region 15 to 25 nt downstream, but not upstream, of the HDV amber/W editing site significantly increases editing [9]. Here, we created mutations at various sites located opposite the HDAg coding region with the goal of increasing the length of consecutive base-pairing in the HDV RNA. The data indicate that mutants carrying a 16 or 17 consecutive base-paired antigenomic segment located as far as 114 nt upstream of the amber/W site in their antigenomes could up-regulate HDV-1 amber/W site editing (Figure 1 and Figure 4). We thus propose that the overall size of the RNA structure regulating HDV-1 RNA editing, including regions upstream of the editing site, appears to be larger than previously thought. Furthermore, our data suggest that suboptimal ADAR1-catalyzed amber/W RNA editing and optimal HDAg binding require strict regulation of the distribution of double-stranded regions and internal bulges in the unbranched rod-like structure of the HDV RNA. 

**Figure 5:**
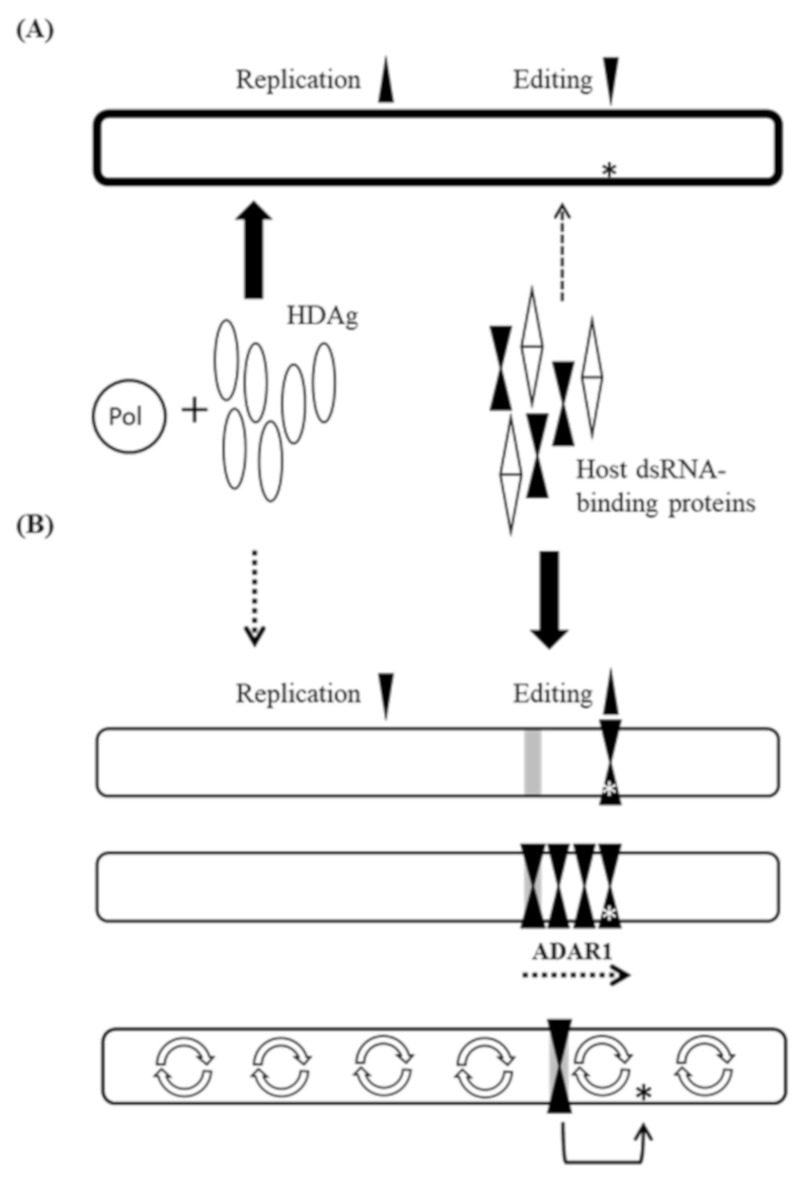
Schematic description of the proposed regulatory mechanism of the HDV-1 amber/W RNA editing. Bold and dashed arrows represent high and low binding affinities, respectively, for the interactions between HDV RNA and various proteins. Star indicates the amber/W editing site. (**A**) Bold oval indicates the HDV RNA with efficient replication ability. The unbranched rod-like structure with proper distribution of duplex segments and bulges is optimal for HDAg binding and suboptimal for ADAR1 binding and amber/W site editing, which favors HDV RNA replication. (**B**) Oval drawn with thin line indicates the HDV antigenomic RNA containing a region of 16 or 17 consecutive bp (gray rectangle). The elongated double-stranded segment excludes HDAg binding and attracts ADAR1 binding (top and middle). Bending or wrapping, as depicted by 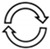, occurs to form high-order structured HDV RNA and to bring ADAR1 and amber/W editing site in close proximity (bottom). Individual steps are described in the text.

Based on our present results and the previous reports [9], we propose a model (Figure 5) that illuminates the consequences of different interactions of various patterns of rod-like structure with host double-stranded RNA binding proteins, including ADAR1, and the viral unbranched rod-like RNA binding protein, HDAg. As shown in Figure 5A, the naturally occurring HDV RNA interacts with HDAg preferentially, which reduces the access of ADAR1 and subsequent amber/W editing [14,15] and recruits replication complexes to initiate HDV RNA replication. Our data suggest that HDV mutants carrying an elongated double-stranded region (16 and 17 bp) disfavor HDAg binding and subsequently favor ADAR1 binding at the amber/W site (Figure 5B, top). Alternatively, this elongated double-stranded region could provide a proper target for ADAR1 binding (Figure 5B, middle). Although the extended double-stranded region itself is not a good substrate for the action of ADAR1, we hypothesize that ADAR1 binds cooperatively to form multimers or moves along the HDV RNA until it reaches the amber/W site, which is present as an A-C mismatch pair with G as the 5′ neighbor [9]. Consequently, this type of HDV RNP fails to support efficient replication (Figure 5B) but promotes RNA editing. Moreover, high-order structured HDV RNA [34] might play an important role not only in virus assembly but also in RNA replication and ADAR1-mediated amber/W editing. HDV RNA in the RNP complexes is condensed by bending or wrapping, which might lead to the “distant” structural elements, such as long consecutive base-paired regions which could attract ADAR1, close to the amber/W editing site, to promote HDV RNA editing (Figure 5B, bottom).

Collectively, our mutagenesis data (m1, m8~m14, and m2-15) suggest that the RNA structure of WT clone I could render the HDV genome able to tolerate the mutations that accumulate during replication while also restricting the editing efficiency, which is important for viral survival. The other mutants containing 16 or 17 consecutive base-paired regions on their antigenomes exhibited promotion of amber/W editing. We examined the GC contents of the experimentally designed duplex regions but did not find evidence that this parameter played a significant role in the level of RNA replication or the amber/W editing rate. We do not yet know whether the extended base-pairing on the HDV circular RNA reduces the stability of the processed RNA and/or disrupts yet-unidentified cis-elements required for replication. It also remains unknown whether different HDV RNA structural patterns could attract different kinds of cellular proteins to form a variety of HDV RNP complexes that differ in their abilities to trans-activate viral RNA synthesis. Although a higher level of large HDAg would favor virion assembly (Figure 3C), the edited genomes packaged into virions could not express small HDAg. As such expression is required for HDV RNA synthesis [11], such particles are not expected to be infectious. This suggests that virus propagation could be negatively affected by an excessive base-pairing that improves the efficiency of amber/W editing, and seems to explain why the predicted secondary structures of the naturally occurring RNA examined in Table 1 consistently exhibited no more than 15 consecutive bp on their genomes.

Clearly, our findings improve our understanding of the interplay of bulges and consecutive bp in HDV RNA-protein (both viral and cellular) interactions, which in turn regulates RNA synthesis and amber/W editing. Our findings also have implications for the selection of naturally occurring HDV RNA. A recent report indicated that HDV activates the interferon response in hepatocytes, but its replication is resistant to self-induced innate immune responses [35]. This indicates that the escape of innate immunity is not the major selection criteria for HDV RNA structure. Here, we suggest that the disruption of HDAg binding, the accumulation of replication-incompetent HDV RNA, and the overexpression of large HDAg collectively contribute to the elimination of HDV mutants with extensive base-paired segments on their RNA. 

## Figures and Tables

**Table 1:** The lengths of the longest base-paired segments in the genomes of 41 HDV-1 strains.

Consecutive bp ^†^	Accession no.			
9	AM779576			
10	AB118849	HQ005365	HQ005369	HQ005370
	AM779580	KF660600	HQ005367	HQ005371
	HQ005372	M58629	M28267	
11	AM902165	AM902170	JX888099	AF425644
	X77627	D01075	AM779579	AM902177
	AM902179	M21012		
12	AJ000558	AM779574	AM902164	AM902167
	JX888101	JX888112	AM779594	HM046802
	M92448	X85253	AF098261	
13	U81988	AM902166		
14	JX888108	HQ005364	AM902174	
15	U81989	HQ005366	HQ005368	

^†^ The predicted secondary structure was obtained using the program RNA structure (version 3.7) [29].

## Supplementary Materials

The following are available online at https://www.mdpi.com/1999-4915/11/10/934/s1, Figure S1: Schematic representation of the design and locations of the HDV mutation sites of m5~m14, Figure S2: Schematic representation of the design and locations of the HDV mutants containing an antigenomic RNA segment that forms 16 or 17 consecutive bp, Table S1: List of primers and templates used for PCR-based mutagenesis of the HDV genome.
