# Supplementary material for: Structural Pattern Differences in Unbranched Rod-Like RNA of Hepatitis Delta Virus Affect RNA Editing
Source: Viruses. 2019 Oct 11;11(10):934. doi: 10.3390/v11100934 (PMC6832723; doi:10.3390/v11100934)
Supplement: Supplementary file 1 [file viruses-11-00934-s001.pdf]

**Supplementary Materials**

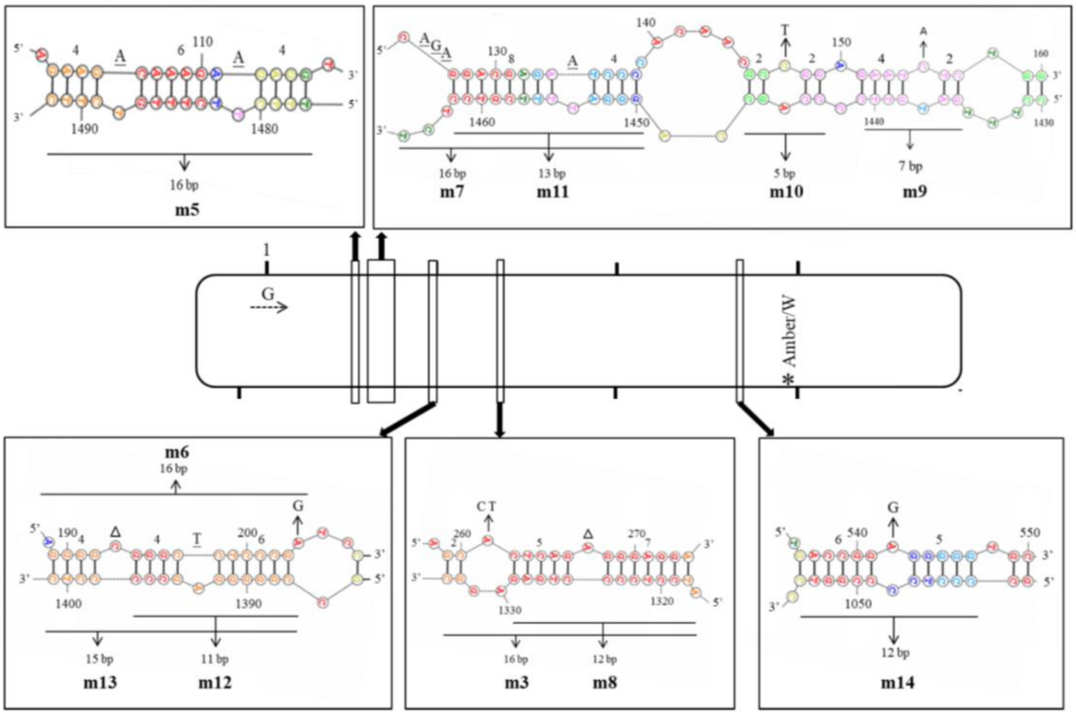


**Figure S1.** Schematic representation of the design and locations of the HDV mutation sites of m5~m14. The RNA structure was predicted using RNAstructure. Partial unbranched rod-like RNA structures of the genomic HDV RNAs containing the mutation sites are shown. cDNA sequences are shown. Color annotation was performed according to base-pairing probability as described for Figure 1A. Base insertions and deletions are underlined and indicated with triangles, respectively. Base substitution is shown above the arrow and vertical lines indicate the formation of base pairing in the HDV circular genome.

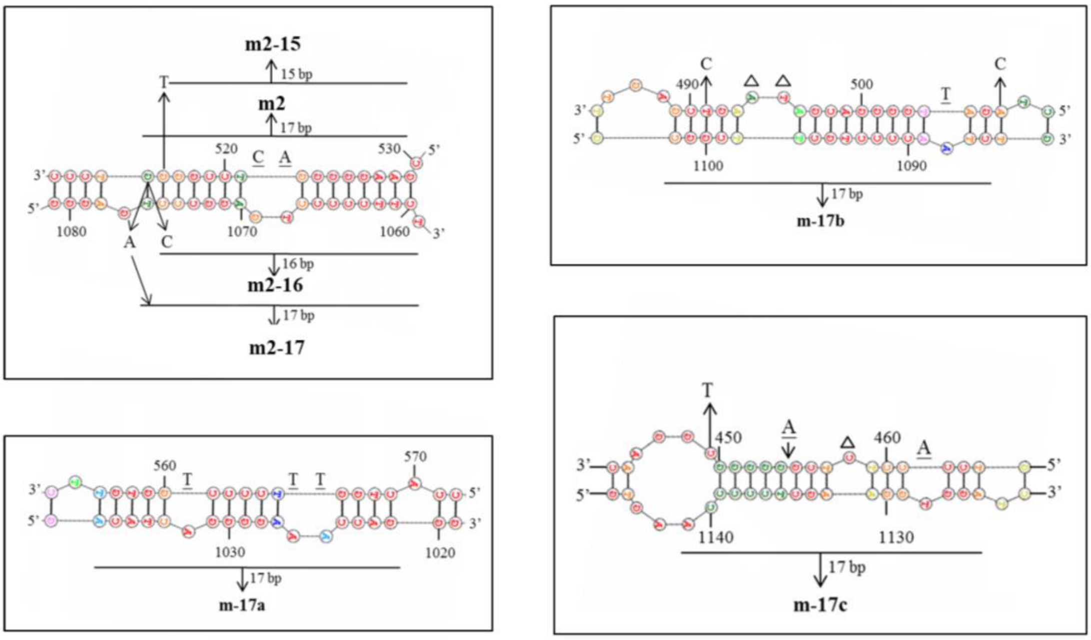


**Figure S2.** Schematic representation of the design and locations of the HDV mutants containing an antigenomic RNA segment that forms 16 or 17 consecutive bp. Partial unbranched rod-like RNA structures of the antigenomic HDV RNAs containing the mutation sites are shown. cDNA sequences are shown. Symbols and description are as described for Figure S1.

**Table S1.** List of primers and templates used for PCR-based mutagenesis of the HDV genome

| Primer name | Sequence (5′→3′) † | Position (nt) | Template (pGEM-4Z-) |
|---|---|---|---|
| m1 | GTAGGAGTAAGACCATAGCG- -AGGAGGAAGATGCTAGGAGTAGGG | 345-389 | D1I |
| m2 | ATCTAGCGGGACCCCGGAGTCCCCCTTCGAAAGTGACC | 504-539 | D1I |
| m3 | GCCCGAAGGGTTGAGTAGC**CT**CTCAG-GGGAGGAATCCACTCGGAG | 242-286 | D1I |
| m4 | CGGGCCTCCCGATCCGAAGGGGGCCCGAATCCCAGATCTGGAGAGCAC | 193-237 | D1I |
| m5 | GAAACTACTCCCAAGAAGACAAAGAAGAGGTCTTAGGAAGCG | 88-127 | D1I |
| m6 | GG-GGGCTCTCCCG**G**TCCGAGGGGCCCAATC | 191-220 | D1I-m13 |
| m7 | GAGAGGTCTTAGGAAGCAGAGGACGAGAATCCCCAC | 110-141 | D1I-m11 |
| m8 | GGGTTGAGTAGCACTCAG-GGGAGGAATCCACTC | 249-282 | D1I |
| m9 | CAACGCCGGAGAAT**A**TCTGGAAGGGGAAAG | 141-170 | D1I |
| m10 | GATCCCCACAACGC**T**GGAGAATCTCTGGAA | 133-162 | D1I |
| m11 | CTTAGGAAGCGGACGAGAATCCCCACAACGCCG | 117-148 | D1I |
| m12 | GGAAGAAAAAGGGGCGGGCTCTCCCGATCCGAGGGG | 179-213 | D1I |
| m13 | GGAAGGTGGAAGAAAAAGGGG-GGGCTCTCCCGATCCGAGGGG | 172-213 | D1I-12m |
| m14 | CCTTCGAAAGTGACCGG**G**GGGGGTGCTGGGAACAC | 525-559 | D1I |
| m2-15 | CGTCCCCATCTAGCGGGAC**A**CCGGAGTCCCCCTTCG | 497-530 | D1I-m2 |
| m2-16 | CTTATCGTCCCCATCTAGCGGGA**G**CCCGGAGTCCCCCTTCG | 492-530 | D1I-m2 |
| m2-17 | CTTATCGTCCCCATCTAGCGGGA**T**CCCGGAGTCCCCCTTCG | 492-530 | D1I-m2 |
| m-17a | CGGAGGGGGTGCTGGGAACACCAGGGGAAACCAGTGGAGCCATGGGATGC | 539-585 | D1I |
| m-17b | GCTTATCCCGGGGAACTCG**G**CT- -TCGTCCCCAATC**G**AGCGGGACCCCGGAC | 472-522 | D1I |
| m-17c | GGCTAGCCGGTGGGTGTTCC**A**CCCCCTCGA-AGGTGGACGAGTGAGGCTTATCCCG | 429-482 | D1I |

†Base insertions and deletions are underlined and indicated with bars, respectively, while base substitutions are shown in bold.
